# Denitrification and the challenge of scaling microsite knowledge to the globe

**DOI:** 10.1002/mlf2.12080

**Published:** 2023-09-28

**Authors:** G. Philip Robertson

**Affiliations:** ^1^ W. K. Kellogg Biological Station Michigan State University Hickory Corners Michigan USA; ^2^ Department of Plant, Soil, and Microbial Sciences Michigan State University East Lansing Michigan USA; ^3^ Great Lakes Bioenergy Research Center Michigan State University East Lansing Michigan USA

**Keywords:** ecosystems, microsites, N_2_, N_2_O, modeling

## Abstract

Our knowledge of microbial processes—who is responsible for what, the rates at which they occur, and the substrates consumed and products produced—is imperfect for many if not most taxa, but even less is known about how microsite processes scale to the ecosystem and thence the globe. In both natural and managed environments, scaling links fundamental knowledge to application and also allows for global assessments of the importance of microbial processes. But rarely is scaling straightforward: More often than not, process rates in situ are distributed in a highly skewed fashion, under the influence of multiple interacting controls, and thus often difficult to sample, quantify, and predict. To date, quantitative models of many important processes fail to capture daily, seasonal, and annual fluxes with the precision needed to effect meaningful management outcomes. Nitrogen cycle processes are a case in point, and denitrification is a prime example. Statistical models based on machine learning can improve predictability and identify the best environmental predictors but are—by themselves—insufficient for revealing process‐level knowledge gaps or predicting outcomes under novel environmental conditions. Hybrid models that incorporate well‐calibrated process models as predictors for machine learning algorithms can provide both improved understanding and more reliable forecasts under environmental conditions not yet experienced. Incorporating trait‐based models into such efforts promises to improve predictions and understanding still further, but much more development is needed.

## INTRODUCTION

Microbes are responsible for many of the life‐sustaining processes that enable life on Earth. We know a great deal about the most crucial microbial processes at a fundamental metabolic and cellular level, both in vitro and in situ, but as we move away from the microscale, our knowledge becomes more diffuse: in few cases do we have the knowledge to scale processes to entire ecosystems, landscapes, and the globe. Yet, it is at these larger scales where knowledge may be most needed: understanding the regional and global impacts of environmental change—and the potential for mitigating those impacts—requires the ability to scale processes with regional and global impact to regions and the globe.

**Figure 1:**
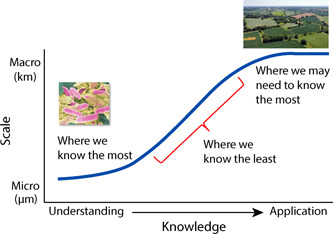
Microbial knowledge across scales. While we have the greatest fundamental knowledge at the microscale (left), it is the macroscale at which knowledge is often needed for decision‐making.

Scaling is thus a growing challenge in microbial ecology. It is useful for conceptualizing our understanding of important processes (Have we identified all the actors and microsites involved?), for evaluating the importance of individual processes (Are there impacts at large scales?), for exploring potentials for intervention (Can we alter large‐scale impacts via local management change?), and for testing our process‐level understanding of microbial outcomes (How well can we model the outcome of a process at large scales?). The challenge is that we know far too little about how to do so well: most of our knowledge is at the scale of microsites, while arguably what we most need to know for understanding global environmental change is at landscape to global scales, leaving a knowledge gap that begs addressing (Figure 1).

Canonical denitrification, the microbial transformation of nitrate or nitrite to nitrous oxide (N_2_O) or dinitrogen (N_2_), provides an ideal model for illustrating the challenge of cross‐scale extrapolation. Complete denitrification involves four major enzyme groups: nitrate reductase, nitrite reductase, nitric oxide reductase, and N_2_O reductase, which sequentially reduce nitrate, nitrite, nitric oxide, and N_2_O to the end product N_2_ ^1^. Not all denitrifiers possess all enzymes, however, such that some may express a different end product or specialize on a different nitrogen substrate. For example, fungal^2^ and some bacterial denitrifiers^3^ lack N_2_O reductase, and denitrifier Clade II N_2_O reducers can directly consume soil pore N_2_O^4^, ^5^.

That said, canonical denitrifiers in general, whether bacterial, archaeal, or fungal denitrifiers, play key roles in regulating the availability of nitrogen to plants, in most ecosystems a limiting nutrient. At the global scale, denitrification largely closes the nitrogen cycle, initiated by N_2_ fixation, keeping the world from becoming awash in toxic levels of nitrogen^6^. And especially important today, canonical denitrification is the major source of atmospheric N_2_O, a major biogenic greenhouse gas with a warming potency ~300 times that of CO_2_, and with an accelerating rate of atmospheric accumulation^7^. That the vast majority of N_2_O in the atmosphere is of microbial origin makes its potential mitigation especially relevant to microbial ecology.

**Figure 2:**
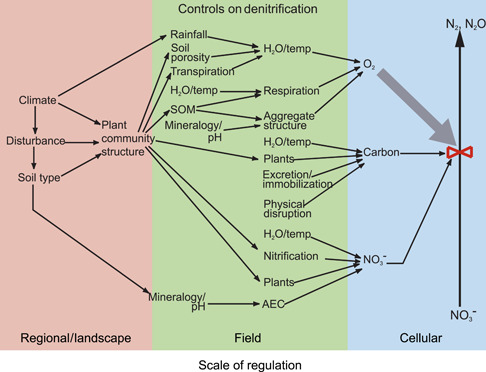
Influence of different environmental factors on canonical bacterial denitrification at different scales. Adapted from Robertson^10^. AEC, anion exchange capacity.

Canonical denitrification also exemplifies scaling challenges because we know at a conceptual level how controls on denitrification vary with scale. Tiedje^8^, ^9^ described a conceptual model for environmental controls on bacterial denitrification in soil and its production of N_2_O and N_2_ that ranged from cellular to regional and global levels (Figure 2). Controls at the cellular level—primarily but not exclusively nitrate, oxygen, and carbon—are influenced by higher‐level controls such as water, soil type, and climate acting at successively greater scales. But operationalizing such a model to allow predictions of denitrification rates at different scales has been difficult.

In the pages that follow, I use canonical bacterial denitrification and its production of N_2_O in terrestrial habitats to illustrate the particular challenges of crossing scales for understanding and predicting denitrifier‐derived N_2_O at local to global scales. While there are other microbial processes known to influence N_2_O emissions—notably fungal denitrification and Clade II N_2_O reduction—their importance is either minor or insufficiently known to be major factors in global N_2_O budgets. Three transitions are particularly important: from cells to microsites, from microsites to fields and landscapes, and from landscapes to the globe. Ultimately, our aim should be to link the rate of atmospheric change in N_2_O concentrations to the underlying microbial processes in such a way that we can inform land management policies that contribute to climate change mitigation^11^, ^12^.

## FROM CELLS TO MICROSITES

It was not until the 1950s' advent of ecosystem N budgets based on mass balance calculations and the availability of ^15^N stable isotope compounds for tracing the fate of N fertilizer in cropping systems^13^ that the potential importance of denitrification in nonhydric soils was recognized. Previously, it was thought that terrestrial denitrification occurred only in wetland and other saturated soils. In the 60 years hence, we have learned that denitrification is a major nitrogen cycle process in most well‐aerated upland soils as well, largely due to the presence of three distinct types of microsites: soil aggregates, plant residue also known as particulate soil organic matter, and soil pores of a particular size. In each of these microsites, the proximal controls on denitrification are relaxed—oxygen stress creates a demand for alternative electron acceptors, while sufficient C and nitrate are available for denitrifiers to respire nitrate to N_2_O and thence perhaps to N_2_ (Figure 2).

**Figure 3:**
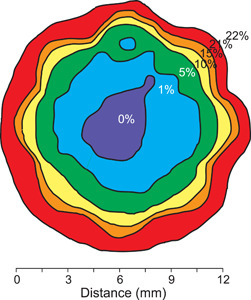
The oxygen profile through a 12 mm soil aggregate. Redrawn from Sexstone et al.^17^

The potential importance of denitrification in soil aggregates was predicted in 1980 by diffusion models^14^, ^15^ that predicted aggregate interiors sufficiently anaerobic to favor denitrifiers. Soil aggregates are comprised in general of soil mineral and organic particles held together with biologically derived polysaccharides^16^, and range in size by orders of magnitude, from <50 to >2000 μm. A thin surrounding water film usually impedes gas exchange, such that oxygen within the aggregate is consumed faster than it can be replaced by diffusion through the film. Oxygen diffuses through water about 10,000 times more slowly than through air. This results in concentric bands of increasingly lower oxygen concentrations toward the center of the aggregate, as first measured by Sexstone et al.^17^ (Figure 3). This stratification also helps to explain why decomposition is attenuated inside aggregates, leading to soil C accrual^18^, ^19^, ^20^.

**Figure 4:**
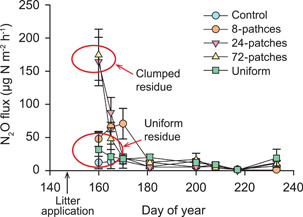
N_2_O emissions from clumped versus dispersed clover litter in a field mesocosm planted to maize. Redrawn from Loecke and Robertson^22^.

A similar phenomenon likely powers denitrification in small pieces of soil organic matter more often called particulate organic matter (POM). For example, in a 1987 paper, Parkin^21^ segmented 15 cm soil cores into progressively smaller portions to show, in one typical case, that 85% of the core's denitrification capacity could be isolated to a single leaf fragment of *Amaranthus* sp. More recently, Loecke and Robertson^22^ documented a similar finding for ^15^N‐labeled clover residue, where the same amount of litter clumped into fewer patches in a succeeding maize crop produced vastly different amounts of N_2_O (Figure 4). Again, microsites with ample carbon and nitrogen protected from oxygen were responsible for much of the soil's denitrification capacity.

**Figure 5:**
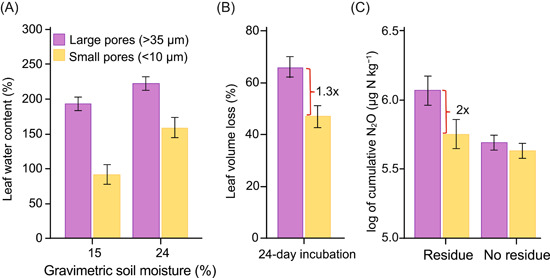
Pore size effects on leaf residue moisture content, decomposition rate, and N_2_O emissions. (A) Leaf residue water content in particles adjacent to large versus small soil pores in soil cores at 15% and 24% gravimetric moisture. (B) Decomposition of residue after 24‐day incubation. (C) N_2_O production from residue adjacent to large pores. 1.3× and 2× refer to the effect magnitudes. Redrawn from Kravchenko et al.^23^

The converse of soil aggregates—soil pores—is a surprising third type of denitrification hot spot in upland soils. Using X‐ray microtomography, Kravchenko et al.^23^ imaged the interior of 3 cm^3^ intact soil cores to reveal differences in soil pore structures among soils planted to annual versus perennial crops, with pores in the perennial crops more connected and continuous and with a lower proportion of large pores. The importance of these pore size differences for decomposition and denitrification became clear in subsequent experiments, which showed POM more likely to absorb water from adjacent large pores than from small pores: in pores >35 μm in size, POM absorbed water like a sponge—to 200% moisture content—as compared to POM adjacent to smaller pores (Figure 5A). And these differences led to a 30% higher decomposition rate in the larger pores (Figure 5B) and effectively doubled rates of N_2_O production (Figure 5C). In the absence of POM, pore size had no effect. Both of these results confirm the importance of POM hotspots for denitrification in soil and the role of soil pores of a particular size for providing absorbable water to POM particles. Differences among soils in their distributions of water in pores of different sizes help, then, to explain differences in microbial process rates like C processing^24^ and N_2_O production.

What all of these microsites have in common is ample C and nitrate together with low oxygen concentrations converging for some period of time over some proportion of soil volume to produce meaningful fluxes of N_2_O and N_2_. Clearly, we have a good handle on scaling canonical denitrification from cells to microhabitats. Where it gets trickier is scaling from microhabitats to ecosystems.

## FROM MICROSITES TO ECOSYSTEMS

The N_2_O and N_2_ emitted by denitrifiers are highly episodic in all terrestrial ecosystems thus far examined. Likewise, these N gas fluxes tend to be highly localized at scales larger than microsites, perhaps as a function of microsite distributions in soil: measured denitrification rates in intensively sampled field sites are among the most spatially variable of any major C and N cycle process. For example, in a 0.5 ha portion of a southern Michigan, USA, grassland, Robertson et al.^25^ found rates of denitrification that were lognormally skewed, ranging over three orders of magnitude with a coefficient of variation four to five times that for soil respiration and N mineralization (Table 1).

**Table 1:** Rates of denitrification across a 0.5 ha old field in southern Michigan, USA as compared to other C and N cycle processes.

Measure	Mean	SD	CV (%)
N mineralization (μg N cm^−2^ day^−1^)	3.36	1.95	57.9
Nitrification (μg NO_3_^−^‐N cm^−2^ day^−1^)	2.52	1.69	67.0
Denitrification (μg N cm^−2^ day^−1^)	4.73	13.0	275
CO_2_ production (μg C cm^−2^ day^−1^)	55.5	33.7	60.7
pH as [H+] (μmol l^−1^)	5.77	3.27	56.6
Moisture (μg H_2_O cm^−2^)	0.65	0.38	58.7
Soil nitrate‐N (μg NO_3_^−^‐N cm^−2^)	5.90	3.84	65.0

*n* = 201 soil cores. CV, coefficient of variation; SD, standard deviation.

Source: From Robertson et al.^25^

**Figure 6:**
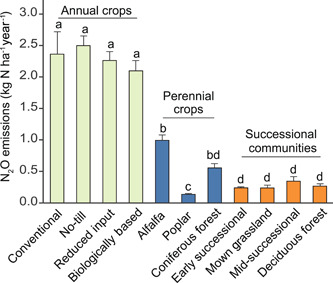
Annual N_2_O fluxes from Michigan, USA ecosystems on the same soil series sampled at weekly to monthly intervals for 25 years. Different lowercases represent significant differences. From Gelfand et al.^28^

Yet, despite such variability, we can often detect differences in rates of denitrification and N_2_O production among ecosystems, especially following disturbance^26^, ^27^, or among different management intensities. For example, in a synthesis of 25 years of flux measurements at the KBS Long‐term Ecological Research site, Gelfand et al.^28^ documented significant, several‐fold differences between an annual crop rotation, whether managed as conventional, no‐till, reduced input, or biological based systems; perennial crops both N fixing and non‐N fixing; and unmanaged grasslands and forests (Figure 6). Consistent long‐term sampling like this is rare but allows greater confidence in the relative magnitude of flux differences that might not be consistent year to year. In this case, the similarity in fluxes between the conventional system, which received synthetic N fertilizer, and the organic system, which received exogenous N only from N‐fixing cover crops, was particularly surprising, underscoring the fact that it is the amount of N cycling through the system that matters most to annual fluxes rather than the source of N. Subsequent analyses^29^ identified denitrifiers rather than nitrifiers as the dominant source of N_2_O emissions.

Despite the long‐term nature of these and other analyses, the annual fluxes represented remain only estimates at best. In situ measurements of N_2_O fluxes at weekly to monthly intervals, even with careful interpolation between sampling events, are really just best guesses—in most cases, we have high confidence only in the relative magnitudes of such fluxes, not the absolute magnitudes. This is because we rarely sample with sufficient temporal intensity to know that we have captured a representative number of flux events. For N_2_O production, temporal variability is usually as extreme as spatial variability and further complicates scaling to larger geographic areas. Short bursts of N_2_O emissions can be responsible for most of an annual flux, especially in intensively managed systems amended with exogenous N from fertilizers, compost, or leguminous cover crops.

**Figure 7:**
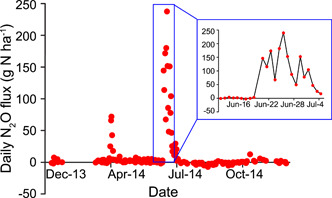
Short bursts of N_2_O fluxes can drive annual emissions. Daily N_2_O fluxes in a Michigan, USA maize cropping system fertilized at planting (25 kg N ha^−1^) and then side‐dressed 6 weeks later (150 kg N ha^−1^) are shown. Data from Saha et al.^30^

**Figure 8:**
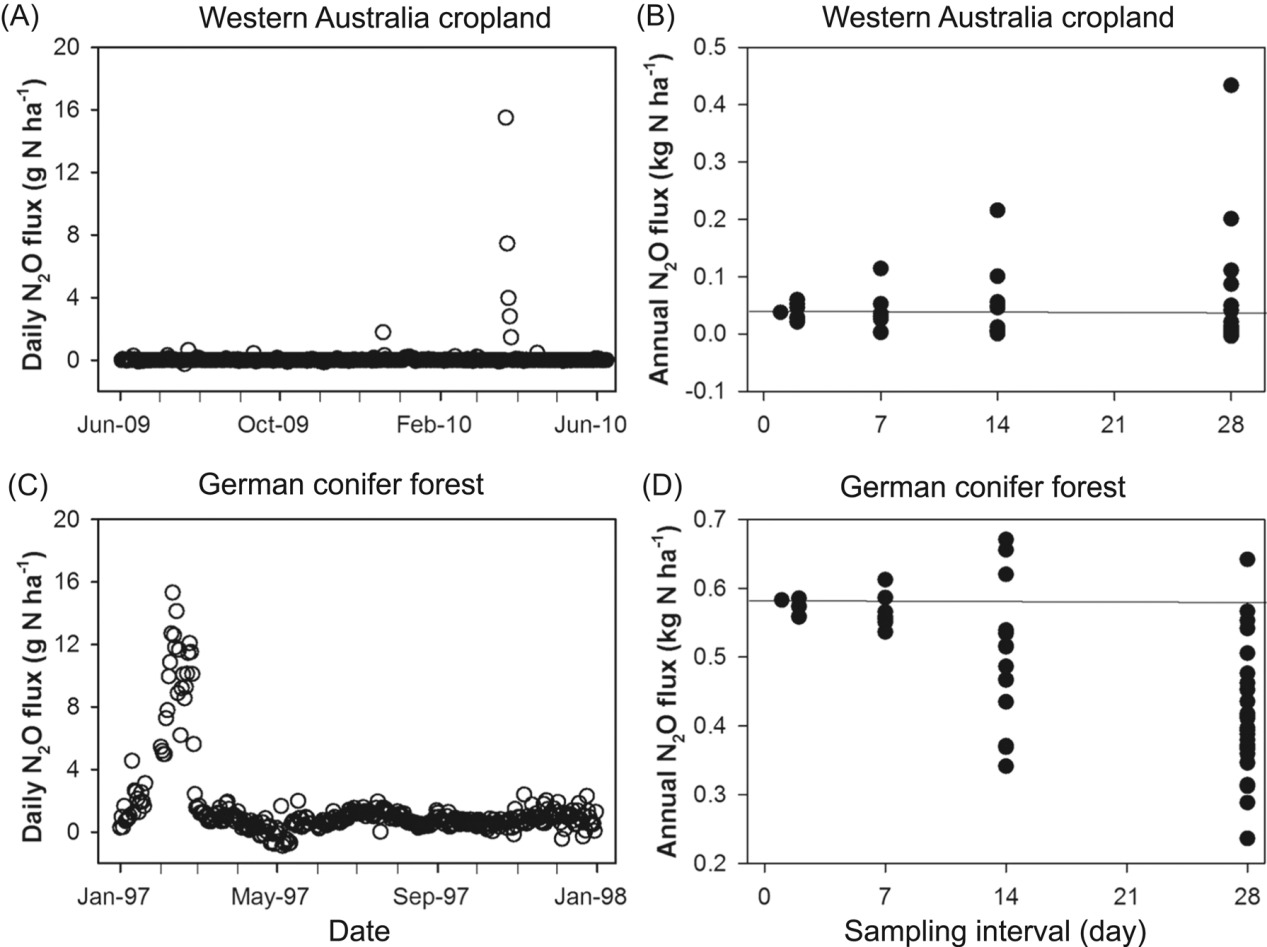
N_2_O response to fertilizer levels. Daily N_2_O fluxes in an Australian wheat field (A), a German forest (C), and respective annual fluxes estimated by subsampling the data in (A) and (C) at successively smaller daily intervals (B, D). From Barton et al.^32^

Figure 7 illustrates the challenge: high‐frequency measurements of fertilized cropland such as those for a maize field in the upper US Midwest typically reveal extraordinarily high fluxes following management events like N fertilization that persist for only a short while, in this case for only a two 2‐week period annually. Temporally intensive measurements such as these are becoming more common with the advent of automated flux chambers that sample at subdaily intervals^31^, and automated measurements in targeted ecosystems are revealing the importance of episodic emissions in even less intensively managed systems such as dryland wheat farming in Western Australia and conifer forests in southern Germany (Figure 8A,C). Barton et al.^32^ showed that in these and other systems for which there are high‐frequency measurements, more frequent sampling—on the order of 3−7‐day intervals throughout the year—may be necessary to estimate annual fluxes with useful precision (Figure 8B,D).

This kind of knowledge is important because it is the annual fluxes that we need to build credible global N_2_O budgets^33^ and to evaluate whether management interventions to mitigate N_2_O will have significant effects at the landscape and global scales.

## FROM ECOSYSTEMS TO THE GLOBE

Estimates of the global N_2_O budget appear less out of balance now than in the 1990s, when less than half of known atmospheric sinks (14.1 Tg N_2_O‐N yr^−1^) could be ascribed to known sources^34^, ^35^. More recent efforts^33^ combining bottom‐up (mainly inventory and statistical extrapolations) and top‐down (atmospheric inversion modeling) approaches provide greater agreement and identify fertilized soils as the main source of the 2% per decade increase in the atmosphere's N_2_O burden.

**Figure 9:**
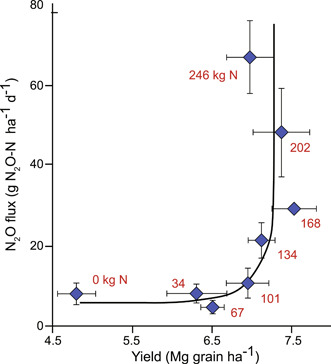
Soil N_2_O fluxes are exquisitely sensitive to nitrogen fertilizer inputs. Once N inputs exceed crop N needs (~130 kg N ha^−1^), N_2_O fluxes are exponentially greater in this maize‐based cropping system in the US Midwest. Mg, megagram. From McSwiney and Robertson.^38^

The agreement between bottom‐up and top‐down approaches is not to say that we are accurately estimating cropland N_2_O emissions: bottom‐up IPCC budgets continue to rely mainly on the linear relationship between N inputs and N_2_O emissions as identified in a 1996 cross‐ecosystem analysis of different fields fertilized at various rates^36^. The slope of this relationship is the basis for the IPCC's Tier I 1.25% emission factor^37^. Yet, more recent studies of individual fields fertilized at different rates suggest that a 1.25% emission factor often severely underestimates emissions at input rates that exceed crop N demand^38^, ^39^, ^40^, ^41^, common in the Global North and elsewhere^42^ due to insufficiently precise or insufficiently followed on‐farm N recommendations^43^ and because of in‐field spatial variability^44^. Within‐field variability plays a role because in only a portion of evenly fertilized fields are yields consistently high; everywhere else in the field, lower productivity will result in some larger amount of N remaining in the soil available to denitrifiers. Once inputs exceed crop N needs, denitrifiers and other N_2_O producers no longer compete with plants for available N, and N_2_O emissions increase exponentially (Figure 9).

Despite the generality of this N_2_O‐fertilizer response^45^, no process‐level N_2_O models can currently reproduce it, likely because we do not yet know its microbial basis, which probably is more complex than simple resource availability. Process‐based N_2_O models such as DayCent^46^ and DNDC^47^ are usually developed from microcosm incubations and infrequent chamber‐based field responses to individual environmental factors, so this is probably to be expected. This approach necessarily (and by design) simplifies the complex biophysical interactions typical of field settings but compromises the models' abilities to make short‐term predictions for sites or experimental conditions for which the model parameters have not been tailored. Moreover, process‐based models do not yet account for new knowledge of microbial processes that affect N_2_O emissions, such as Clade II N_2_O reducers^4^, ^5^.

Thus, when compared with measured data, current process‐based models of N_2_O fluxes do a relatively poor job of predicting daily fluxes in novel sites—in one synthesis with only 20% accuracy in the 15 cropping system studies for which the models had not been previously tuned^30^. Likewise, an ensemble of 24 process‐based N_2_O models showed equally large uncertainties^48^. This limits their utility for predicting short‐term impacts of management change that might mitigate N_2_O emissions, and by extension, their ability to predict annual fluxes with certainty.

**Figure 10:**
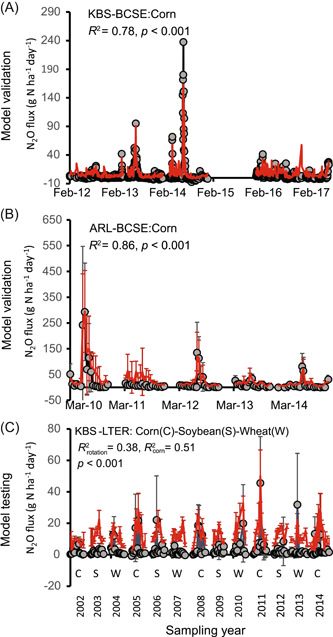
Machine learning predictions for N_2_O emitted from continuous maize cropping systems. Alfisol (A) and Mollisol (B) soils that were used to train and validate the model and for a naïve site on an Alfisol oil (C). From Saha et al.^30^

**Figure 11:**
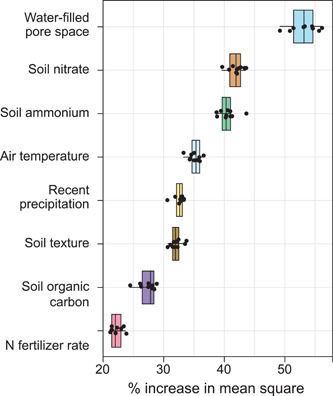
Environmental predictors in rank order for estimating N_2_O fluxes in Figure 10. From Saha et al.^30^

The greater power of machine learning approaches for predicting short‐term fluxes may resolve some of the precision missing from process‐level models. Saha et al.^30^ used data from automated chambers (~3000 subdaily fluxes from a continuous maize system) together with conventional nonautomated static chambers to train a machine learning model capable of predicting daily fluxes with ~50% accuracy for a completely novel site with a different rotation (Figure 10). This is a step in the right direction—two to three times better accuracy than untrained process‐level models, but of course, machine learning models are statistical so they cannot predict fluxes under novel conditions, that is, fluxes that exceed the bounds of the training data. Nor can they be used to test our understanding of key process‐level interactions. That said, they do have the additional advantage of identifying the factors that best predict model outcomes, in this case, water‐filled pore space, soil inorganic N as predicted by a process‐based model, temperature, and precipitation (Figure 11). Hybrid models that use machine learning to predict N_2_O coupled to process‐based models to estimate more readily predicted factors like N pools^30^ may be a promising way forward.

**Figure 12:**
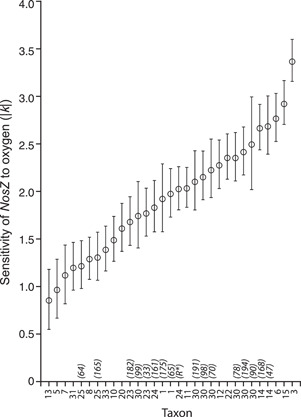
The sensitivity of *nos*Z to oxygen for 31 denitrifier taxa isolated under conditions that favor canonical denitrification from adjacent management systems in Michigan, USA. From Cavigelli and Robertson^55^.

Additionally, however, 30 years of denitrification research suggests that better predictions of denitrification and N_2_O fluxes may require incorporating population or even genome‐level biological traits. Identifying the distribution of life history traits that influence N_2_O production, and incorporating information about the presence of these traits into microbial models of ecosystem functioning, as Malik et al.^49^ did for carbon acquisition strategies and others^50^, ^51^, ^52^, ^53^, ^54^ for decomposition and soil carbon change, could go far toward providing the precision needed for more credible N_2_O fluxes. Cavigelli and Robertson^55^, for example, showed that denitrifiers from different sites on the same soil series differ in their mole ratio (N_2_O:N_2_) response to low oxygen, with some denitrifiers producing mainly N_2_O and others producing mainly N_2_, and a fourfold range overall (Figure 12). That they used isolates grown under conditions known to favor canonical denitrification—and thus represent only a fraction of likely denitrifier diversity in these soils—only serves to underscore the wide range of physiological responses inherent in natural populations. Note that this approach does not involve the inclusion of detailed genomic data in such models, but rather the relative importance of different life history traits as revealed, in part, by genomic data.

Are we ready to incorporate such traits into our N_2_O modeling efforts? Not yet, though capturing traits such as the distribution of *nosZ* gene clades as revealed by genomic analyses and then relating them to functional activity (i.e., traits) under different soil conditions may—coupled with machine learning—allow us to better scale denitrification effectively. Such an approach would be analogous to that used to model soil carbon change in response to global change factors such as warming or nitrogen enrichment. Wieder et al.^50^, ^56^, for example, showed how inclusion in their MIMICS model of copiotrophic and oligotrophic microbes as two different C pools can better capture soil warming responses as compared to conventional single microbial pool models, though challenges remain^57^.

## CONCLUDING REMARKS

To summarize, three points follow. First, scale matters: Both for linking fundamental understanding to application (Figure 1), and for scaling processes to planetary domains to assess the global importance of a process and what might be gained (or lost) when microbial populations are altered at local scales, whether intentionally or inadvertently.

Second, scaling is seldom straightforward. Rarely can simple multiplication of an average rate for an average period produce truly robust results at large scales. Understanding the full range of responses to key environmental controls acting at different spatial (Figure 2) and temporal (Figures 7 and 8) scales seems crucial for addressing questions at progressively greater scales.

And third, we need better quantitative models to better scale. By themselves, process‐level models can be sufficient for very cosmopolitan or very discrete microbial processes, and can be useful everywhere for narrowing our process‐level understanding of microbial process rates. Machine learning may improve predictability and more readily identify best predictors of processes that evade accurate prediction by quantitative models, and thus can be highly informative, but machine learning cannot be relied upon to forecast process rates into novel futures. Hybrid models that include both process‐level and machine learning algorithms might better predict existing rates, as well as forecast future rates, and inclusion of working life history traits in these models might be particularly fruitful, but it is still early days yet.
